# Explainable COVID-19 Detection Using Chest CT Scans and Deep Learning

**DOI:** 10.3390/s21020455

**Published:** 2021-01-11

**Authors:** Hammam Alshazly, Christoph Linse, Erhardt Barth, Thomas Martinetz

**Affiliations:** 1 Institute for Neuro- and Bioinformatics, University of Lübeck, 23562 Lübeck, Germany; linse@inb.uni-luebeck.de (C.L.); barth@inb.uni-luebeck.de (E.B.); martinetz@inb.uni-luebeck.de (T.M.); 2 Mathematics Department, Faculty of Science, South Valley University, Qena 83523, Egypt

**Keywords:** coronavirus, COVID-19 detection, SARS-CoV-2, explainable deep learning, feature visualization

## Abstract

This paper explores how well deep learning models trained on chest CT images can diagnose COVID-19 infected people in a fast and automated process. To this end, we adopted advanced deep network architectures and proposed a transfer learning strategy using custom-sized input tailored for each deep architecture to achieve the best performance. We conducted extensive sets of experiments on two CT image datasets, namely, the SARS-CoV-2 CT-scan and the COVID19-CT. The results show superior performances for our models compared with previous studies. Our best models achieved average accuracy, precision, sensitivity, specificity, and F1-score values of 99.4%, 99.6%, 99.8%, 99.6%, and 99.4% on the SARS-CoV-2 dataset, and 92.9%, 91.3%, 93.7%, 92.2%, and 92.5% on the COVID19-CT dataset, respectively. For better interpretability of the results, we applied visualization techniques to provide visual explanations for the models’ predictions. Feature visualizations of the learned features show well-separated clusters representing CT images of COVID-19 and non-COVID-19 cases. Moreover, the visualizations indicate that our models are not only capable of identifying COVID-19 cases but also provide accurate localization of the COVID-19-associated regions, as indicated by well-trained radiologists.

## 1. Introduction

Coronavirus disease 2019 (COVID-19) is an infectious disease caused by the new coronavirus named severe acute respiratory syndrome coronavirus 2 (SARS-CoV-2). The virus is highly contagious and can be transmitted by direct and/or indirect contact with infected people through respiratory droplets when they sneeze, cough, or even talk [1,2,3]. Currently, the real-time polymerase chain reaction (RT-PCR) test is the standard reference for confirming COVID-19, and with the rapid increment in the number of infected people, most countries are encountering a shortage in testing kits. Moreover, RT-PCR testing has high turnaround times and a high false negative rate [4]. Thus, it is highly desirable to consider other testing tools for identifying COVID-19-contaminated patients to isolate them and mitigate the pandemic’s impact on the lives of many people.

Chest computed tomography (CT) is an applicable supplement to RT-PCR testing and has been playing a role in screening and diagnosing COVID-19 infections. In recent studies [5,6], the authors manually examined chest CT scans for more than a thousand patients and confirmed the usefulness of chest CT scans for diagnosing COVID-19 with high sensitivity rates. In some cases, the patients initially had negative PCR tests; however, confirmation was based on their CT findings. Moreover, chest CT screening has been recommended when patients show symptoms compatible with viral infections, but the results of their PCR tests are negative [5,7]. Nevertheless, diagnosing COVID-19 from chest CT images by radiologists takes time, and manually checking every CT image might not be feasible in emergency cases. Therefore, there is a need for automated detection tools that exploit the recent advances in artificial intelligence (AI) and deep learning along with the availability of CT images to build AI-based tools to expedite the diagnosis process and prevent further spreading [8].

This paper adopts the most advanced deep convolutional neural network (CNN) architectures, which were top performers in the ImageNet large scale visual recognition challenge (ILSVRC) [9], and presents a comprehensive study for detecting COVID-19 based on CT images. We explored CNN models that have different architectural designs and varying depths to obtain the best performance. Even though we conducted our experiments on two of the largest CT scan datasets available for research, they are still of insufficient size to train deep networks from scratch. An effective strategy to overcome this limitation is to use transfer learning [10], where deep networks trained on visual recognition tasks are utilized to initialize networks for different but related target tasks. Most of the published work that applied transfer learning strategies from deep networks pretrained on the ImageNet dataset [11], followed the strict fixed-sized input predetermined for each network architecture, and resized their input images accordingly. However, we argue that resizing images with different aspect ratios to match a specific resolution can distort the image severely. Consequently, we addressed the problem by placing the images into a fixed-sized canvas determined specifically for each CNN architecture, where the aspect ratio of the original image was preserved. This proved to be a less violating and more effective procedure to achieve better results, as reported in [12]. Moreover, we utilized the layer-wise adaptive large batch optimization technique called LAMB [13], which has demonstrated better performance and convergence speed for training deep networks. The performances of the models were measured quantitatively using accuracy, precision, sensitivity, specificity, F1-score, and the confusion matrix for each model. Our results indicate the ability of our models to achieve state-of-the-art results on the considered datasets.

In order to provide better explainability of the deep models and make them more transparent, we applied two visualization techniques. The first approach was the t-distributed stochastic neighboring embedding (t-SNE) [14], which is a dimensionality reduction and visualization technique for visualizing clusters of instances in a high-dimensional space. The visualizations of the t-SNE embeddings show well-separated clusters representing CT images for COVID-19 and non-COVID-19 cases. The second approach was the gradient-weighted class activation mapping (Grad-CAM) [15], which is a visualization technique for CNN-based models. It provides high-resolution and class-discriminative visualizations that localize the important image regions considered for the model prediction. The Grad-CAM visualizations show how accurately our models localize the COVID-19-associated regions. Overall, this paper exhibits the following contributions:

- A comparative experimental study was conducted on how well advanced deep CNNs trained on chest CT images can identify COVID-19 cases. To this end, we experimented with 12 deep networks that have different architectural designs and varying depths, and provide quantitative and qualitative analyses.
- We propose a transfer learning strategy to fine-tune deep networks using custom-sized inputs determined specifically for each architecture, and utilized the LAMB optimizer for training the networks. Our experimental results prove the effectiveness of our strategy to obtain state-of-the-art performance on the considered CT image datasets. Our best models achieved average accuracy of 99.4% and 92.9%, and average sensitivity rates of 99.8% and 93.7% on the largest datasets of CT images available for research.
- We provide visualizations of the features extracted from different models to understand how deep networks represent CT images in the feature space. The visualizations show well-separated clusters representing the CT images of the different classes, which indicates that our models have learned discriminative features to distinguish CT images of different cases.
- We show discriminative localizations and visual explanations obtained by our models for detecting COVID-19-associated regions in CT images as annotated by expert radiologists.

The rest of the paper is structured as follows. We review the related work in the next section. The considered deep CNN architectures are explained in Section 3. Our transfer learning strategy to learn discriminative representations is described in Section 4. The experimental settings and the results are reported in Section 5. Finally, we draw the main conclusions in Section 6.

## 2. Related Work

This section highlights some relevant work that adopted deep CNNs for building computer-aided diagnosis (CAD) systems to facilitate the interpretation of medical images. The authors in [16] employed different deep CNN architectures, which were pretrained on the ImageNet dataset [11], and fine-tuned them on specific CT scans for thoraco-abdominal lymph node detection and interstitial lung disease classification. Their study indicated the effectiveness of deep CNNs for CADs problems even when training data are limited. In [17], the authors proposed the CheXNet model to detect different types of pneumonia from chest X-ray images. The model consisted of 121-layers and was trained on a large dataset that contained over 100,000 X-ray images for 14 different thoracic diseases. The model showed outstanding detection performance at the level of practicing radiologists.

In the context of the COVID-19 pandemic, extensive research has been conducted to develop automated image-based COVID-19 detection and diagnostic systems [18,19,20,21,22,23,24,25]. We hereafter review the proposed approaches for reliable detection systems based on chest X-ray and CT-scan imaging modalities. These techniques follow either one of the two main paradigms.

On one hand, new deep network architectures have been developed and tailored specifically for detecting and recognizing COVID-19. COVID-Net [26] represents one of the earliest convolutional networks designed for detecting COVID-19 cases automatically from X-ray images. The performance of the network showed an acceptable accuracy of 83.5% and a high sensitivity of 100% for COVID-19 cases. Hasan et al. [27] proposed a CNN-based network named the Coronavirus Recognition Network (CVR-Net) to automatically detect COVID-19 cases from radiography images. The network was trained and evaluated on datasets with X-ray and CT images. The results showed varying accuracy scores according to the number of classes in the underlying X-ray image dataset, and an average accuracy of 78% for the CT image dataset. Further modifications were applied to COVID-Net to improve its representational ability for one specific image modality and to make the network computationally more efficient, as in [28]. Mukherjee et al. [29] proposed a tailored CNN architecture composed of nine layers for detecting COVID-19 positive cases. They trained and tested their network using CT scans and X-rays together. The network obtained promising results compared with existing CNN architectures, and achieved an overall accuracy of 96.28%.

On the other hand, some deep networks have been proposed for similar tasks of automated detection and recognition of COVID-19 cases; however, these networks are based on well-designed and existing CNN architectures, such as ReseNet [30], Xception [31], and Capsule Networks [32]. The authors in [22] adopted transfer learning from deep networks for automatic COVID-19 detection using X-ray images from patients with COVID-19, patients with bacterial pneumonia, and disease-free cases. They reported the best results for the two and three-class classification tasks with accuracies of 98.75% and 93.48%, respectively. Minaee et al. [33] applied transfer learning by fine-tuning four popular pretrained CNNs to identify COVID-19 infection. They experimented on a prepared X-ray image dataset with 5000 chest X-rays. Their best approach obtained average sensitivity and specificity values of 98% and 90%, respectively. Brunese et al. [34] utilized transfer learning by using a pretrained VGG-16 network [35] to automatically detect COVID-19 from chest X-rays. On a dataset collected from different sources with X-rays for healthy and pulmonary diseases, they reported an average accuracy of 97%.

Zhou et al. [36] highlighted the importance of deep learning techniques and chest CT images for differentiating between COVID-19 and influenza pneumonias. The study was conducted on CT images for confirmed COVID-19 patients from different hospitals in China. Their study proved the potential of accurate COVID-19 diagnosis from CT images and the effectiveness of their proposed classification scheme to differentiate between the two pneumonia types. DeepPneumonia [37] was developed to identify COVID-19 cases (88 patients), bacterial pneumonia (100 patients), and healthy cases (86 subjects) based on CT images. The model achieved an accuracy of 86.5% for differentiating between bacterial and viral (COVID-19) pneumonia and an accuracy of 94% for distinguishing between COVID-19 and healthy cases. The authors in [38] used CT images to identify COVID-19 patients from non-COVID-19 people by applying transfer learning from a pretrained DenseNet201 network. The model achieved an accuracy of 96.25%.

Very few studies employed handcrafted feature extraction methods and conventional classifiers. In [39], texture features were extracted from X-ray images using popular texture descriptors. The features were combined with those extracted from a pretrained InceptionV3 [40] using different fusion strategies. Then, various classifiers were used to differentiate between normal X-rays and different types of pneumonia. The best classification scheme achieved an F1-score of 83%. In [41], the authors proposed an approach to differentiate between positive and negative COVID-19 cases using CT scans. Different texture features were extracted from CT images with Gabor filters, and then support vector machines were trained for classification. Their proposed scheme achieved an average accuracy of 95.37%, and a sensitivity of 95.99%.

The above discussion of the related work indicates the prominence of deep learning methods in addressing the task of automated COVID-19 detection. We build on the existing body of published work by using advanced deep networks for detecting COVID-19 using CT images. We conducted experiments on two of the largest CT image datasets and compared the performances of 12 deep networks using standard evaluation metrics. We also provide visualizations for better explainability of the resulting models.

## 3. Deep Network Architectures

This section describes the deep CNN architectures utilized to identify COVID-19 using chest CT scans. These networks are state-of-the-art deep models for image recognition. They differ in their architectural design in order to achieve better representational power and to reduce their computational complexity. In this work we consider the most advanced networks, such as SqueezeNet [42], Inception [40], ResNet [43], ResNeXt [44], Xception [45], ShuffleNet [46], and DenseNet [47].

### 3.1. SqueezeNet

**Figure 1:**
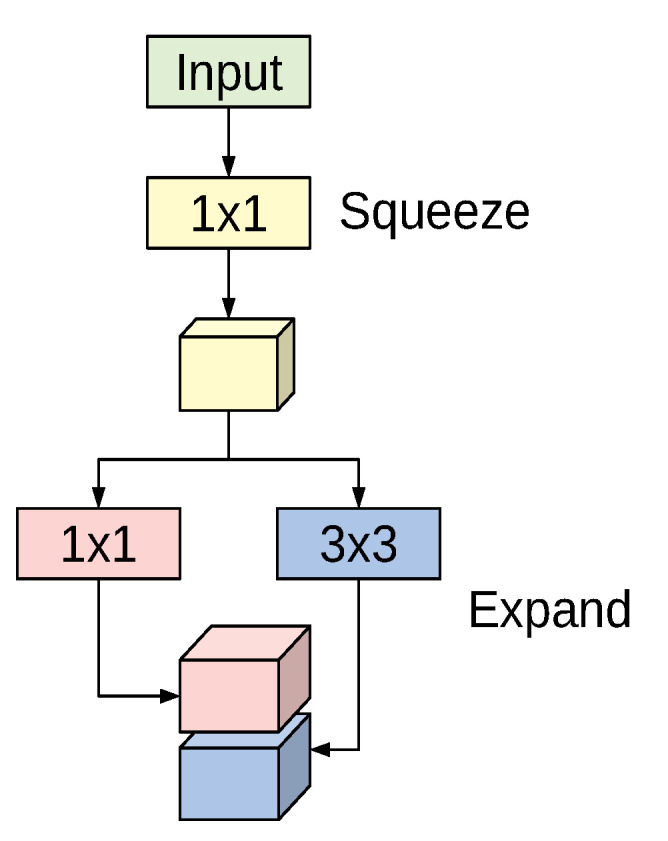
The fire module used in SqueezeNet.

The SqueezeNet architecture is a deep CNN proposed for computer vision tasks with the main concerns on efficiency (having fewer parameters and smaller model size) [42]. The basic building block for the SqueezeNet architecture is the fire module depicted in Figure 1. The module incorporates a squeeze phase and an expand phase. The squeeze phase applies a set of 1×1 filters followed by a ReLU activation. The number of learned squeeze filters is always smaller than the size of the input volume. Consequently, the squeeze phase can be considered as a dimensionality reduction process, and at the same time it captures the pixel correlations across the input channels. The output of the squeeze phase is fed into the expand phase, in which a combination of 1×1 and 3×3 convolutions are learned. The larger 3×3 filters are used to capture the spatial correlations amongst pixels. The outputs of the expand phase are concatenated across the channel dimension and then evaluated by a ReLU activation.

The original paper proposed using *n*, 1×1; and *n*, 3×3 filters in the expand phase, where *n* is 4× larger than the number of filters used in the squeeze phase. The entire SqueezeNet architecture is constructed by stacking conventional convolution layers, max-pooling, fire modules, and an average pooling layer at the end. The model has no fully connected layers. For more details about the number of fire modules for each stage, their order, and numbers of squeeze and expand filters for the different stages, see [42].

### 3.2. Inception

The Inception network is a deep convolutional architecture introduced as GoogLeNet (Inception V1) in 2014 by Szegedy et al. [48]. The architecture has been refined in various ways, such as adding batch normalization layers to accelerate training (Inception V2 [49]), and factorizing convolutions with larger spatial filters for computational efficiency (Inception V3 [40]). We adopt the InceptionV3 model due to its outstanding performance in image recognition and object localization.

**Figure 2:**
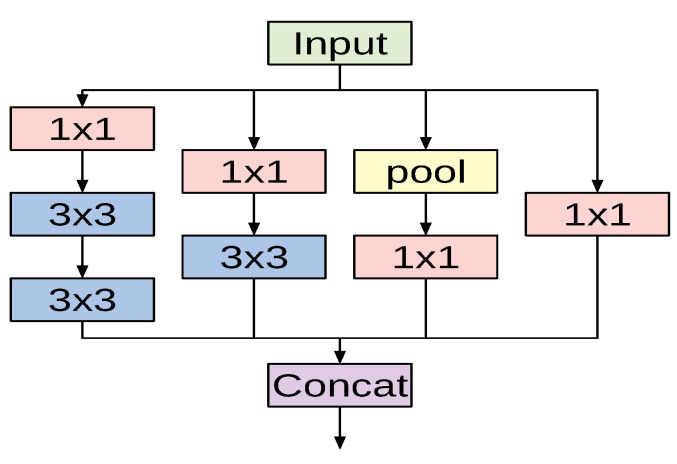
A variant of the Inception module used in InceptionV3 architecture.

The fundamental building block for all Inception-style networks is the Inception module of which several forms exist. Figure 2 shows one variant of the Inception module that is used in the InceptionV3 model. The module accepts an input and then branches into four different paths, each performing a specific set of operations. The input passes through convolutional layers with different kernel sizes (1×1 and 3×3) and a pooling operation. Applying different kernel sizes allows the module to capture complex patterns at different scales. The outputs of all branches are concatenated channel-wise.

The overall architecture of the InceptionV3 network is composed of conventional 3×3 convolutional layers at the early stages of the network, where some of these layers are followed by max-pooling operations. Subsequently, a stack of various Inception modules is applied. These modules have different designs with respect to the number of applied filters, filter sizes, depth of the module after symmetric or asymmetric factorization of larger convolutions, and when to expand the filter bank outputs. The last Inception module is followed by an average-pooling operation and a fully connected layer.

### 3.3. ResNet

Deep residual networks (ResNet) proposed by He et al. in [43], represent a family of extremely deep CNN architectures that won the ILSVRC-2015 challenges for image recognition, object detection and localization [9]. The winning network was composed of 152 layers, which confirms the beneficial impact of network depth on visual representations. However, two major problems are encountered when training networks of increasing depth: vanishing gradients and performance degradation. The authors addressed the problems by adding skip connections to prevent information loss as the network gets deeper.

**Figure 3:**
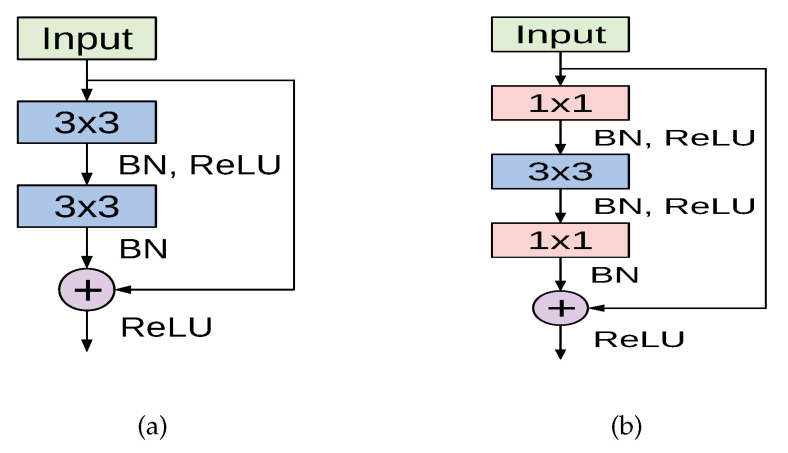
The basic residual module used in ResNet18 (**a**), and the bottleneck residual module utilized in ResNet50 and ResNet101 (**b**), both as introduced in [43].

The cornerstone for constructing deep residual networks is the residual module, of which two variants are depicted in Figure 3. The left path of the residual module in Figure 3a is composed of two convolutional layers, which apply 3×3 kernels and preserve the spatial dimensions. Batch normalization and ReLU activation are also applied. The right path is the skip connection where the input is added to the output of the left path. This variant is used in the ResNet18 model. Another variant of the residual module named the bottleneck residual module is depicted in Figure 3b, in which the input signal also passes through two branches. However, the left path performs a series of convolutions using 1×1 and 3×3 kernel sizes, along with batch normalization and ReLU activation. The right path is the skip connection, which connects the module’s input to an addition operation with the output of the left path. This variant is utilized in ResNet50 and ResNet101 models.

A deep residual network is constructed by stacking multiple residual modules on top of each other along with other conventional convolution and pooling layers. For our experiments we implemented three variants of ResNet: ResNet18, ResNet50, and ResNet101 models. The full configurations and the overall structure of each model are given in [43].

### 3.4. ResNeXt

**Figure 4:**
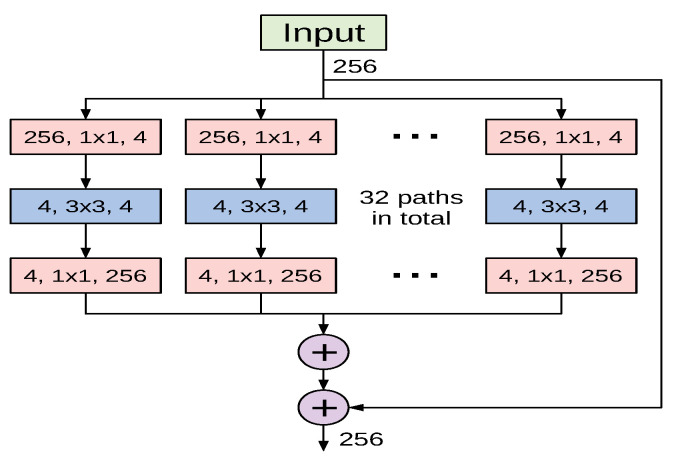
A basic ResNeXt block with cardinality of 32 as proposed in [44].

The ResNeXt architecture proposed in [44] is a deep CNN model constructed by stacking residual building blocks of identical topology in a highly modularized fashion. The ResNeXt architectural design was inspired by the well-designed architectures of ResNet and Inception models. Similarly to the former, it stacks multiple building blocks to construct deeper networks, and it exploits the split-transform-merge strategy of the latter in an expendable manner. Nevertheless, the building blocks of ResNeXt apply an identical set of transformations in all branches, which makes the number of branches an independent hyperparameter to be investigated. The authors referred to the size of the set of transformations as cardinality, and empirically investigated its impact on the network’s representational power. Figure 4 shows a variant of ResNeXt block with a cardinality of 32.

To construct a deep ResNeXt network, conventional convolution and max-pooling layers are used at the beginning of the network and are followed by multiple stages of varying ResNeXt blocks. In our experiments we utilized two variants of ResNeXt: ResNext50 and ReNeXt101. Even though original ResNeXt models used RGB-inputs of 224×224, we decided to use the same input size as for ResNet variants, 349×253, due to similar architectural design.

### 3.5. Xception

Xception is a deep CNN architecture proposed in [45]. It was inspired by the Inception architecture and utilizes the residual connections proposed in ResNet models [43]. However, it replaces the Inception modules with depthwise separable convolution layers. A depthwise separable convolution consists of a depthwise convolution (spatial convolution of 3×3, 5×5, etc.) performed over each channel of an input to map the spatial correlations, followed by a pointwise convolution (1×1) to map the cross-channel correlations.

The Xception architecture depends entirely on depthwise separable convolution layers with a strong assumption that spatial correlations and cross-channel correlations can be mapped separately. The network consists of 36 convolutional layers structured into 14 modules. All modules have residual connections except for the first and last modules. The reader is referred to [45] for a complete description of the model specification.

Due to its superior performance in vision tasks, we adopted the Xception model in our experiments. Even though the original model used an RGB-input of size 299×299, we found that an input size of 327×231 obtains the best results.

### 3.6. ShuffleNet

ShuffleNet is a very computationally-efficient CNN architecture that is mainly designed for mobile devices with constrained computational power [46,50]. The architecture introduces two important operations to significantly reduce the computational cost while maintaining accuracy. The first operation is pointwise group convolutions, which can reduce the computational complexity of the 1×1 convolutions. The second operation consists of shuffling the channels, which assists the information flow across feature channels.

**Figure 5:**
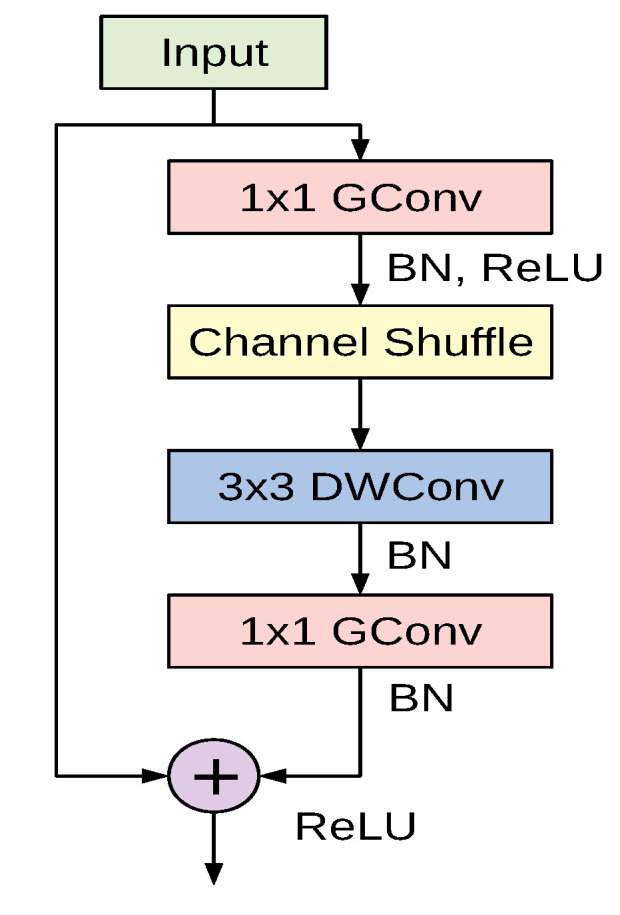
The building unit of the ShuffleNet architecture.

The cornerstone of the ShuffleNet model is the ShuffleNet unit depicted in Figure 5. It is a bottleneck residual module in which the 3×3 convolutional layer is replaced by a 3×3 depthwise separable convolution as in [45]. Additionally, the first 1×1 convolutional layer is replaced by a pointwise group convolution followed by a channel shuffle operation. The second pointwise group convolutional layer is used to retrieve the channel dimension to match the left path of the unit. The overall ShuffleNet network is composed of a stack of these units grouped into three different stages along with other conventional convolution and pooling layers.

In this study we adopted the recent variant of the ShuffleNet architecture. The original model used an RGB-input of 224×224; however, we found that an input resolution of 321×225 works better for the considered datasets.

### 3.7. DenseNet

Densely connected convolutional networks (DenseNets) are a class of CNN architectures introduced in [47] with several compelling characteristics. They alleviate the vanishing gradients problem, foster feature reuse, achieve high performance, consolidate feature propagation, and are computationally efficient. DenseNets modify the shortcut connections from ResNet by concatenating the output of the convolutions instead of summing them up. Thus, the input to the next layer will be the feature maps of all the preceding layers.

**Figure 6:**
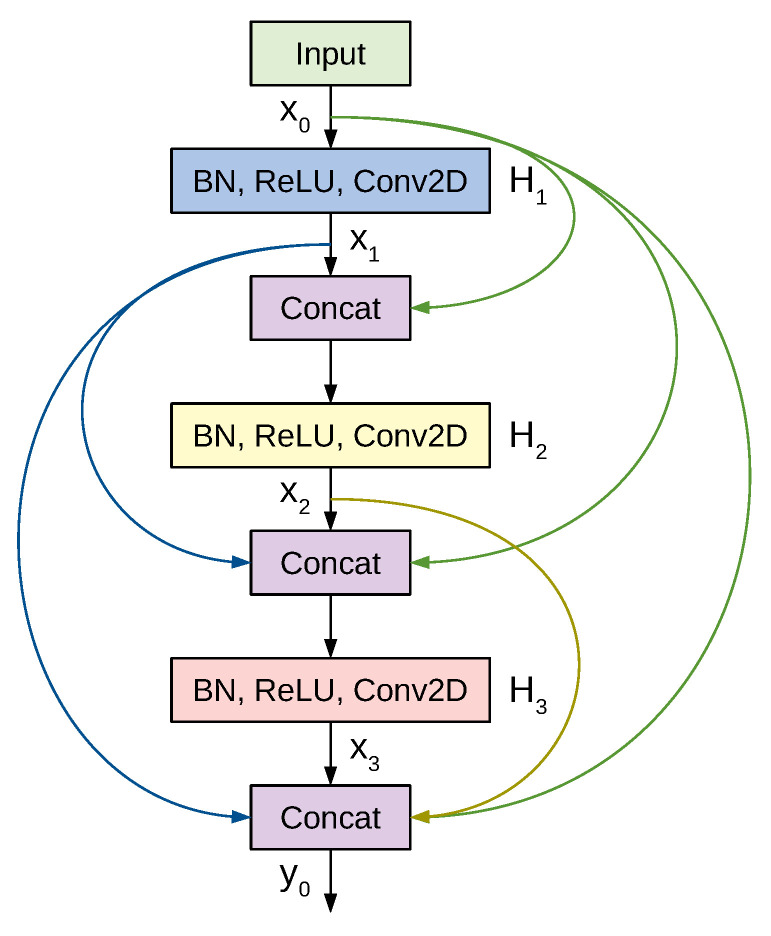
A 3-layer Dense block in DenseNet. The input to each layer is all the previous feature maps.

Figure 6 shows a 3-layer dense block where each layer performs a set of batch normalization, ReLU activation, and 3×3 convolution operations. Previous feature maps are concatenated and provided as the input to a subsequent layer, which then generates *k* feature maps, where *k* is a newly introduced hyper-parameter denoted as the growth rate. Thus, if the input to layer $x_{0}$ is $k_{0}$, then the number of feature maps at the end of a 3-layer dense block is $3\times k+k_{0}$. To prevent the number of feature maps from increasing too rapidly, DenseNet introduces a bottleneck layer with 1×1 convolution and 4×k filters. To tackle the difference in the feature map sizes when transitioning from a large feature map to a smaller one, DenseNet applies a transition layer made of 1×1 convolution and average pooling.

A deep DenseNet is constructed by stacking multiple dense blocks with transition layers. Conventional convolution and pooling layers are used at the beginning of the network. Eventually the output is pooled by global average pooling, flattened, and passed to a softmax classifier. For our study we experimented with three DenseNet variants: the 121-layer, the 169-layer, and the 201-layer architectures. The original models used an RGB-input of 224×224; however, we found that an input size of 349×253 achieves better results for images from the used datasets.

Table 1 summarizes the important characteristics of the adopted deep CNN models. Those include the square-sized input for each network, our proposed custom-sized input, trainable parameters in millions, number of layers, and model size in megabytes.

## 4. Transfer Learning

Transfer learning is an effective representation learning approach in which the networks trained on abundant numbers of images (millions) are used to initialize the networks for tasks for which data are scarce (a few hundred or thousand images). In the context of deep learning there are two common strategies to apply transfer learning from pretrained networks: feature extraction and fine-tuning [51,52]. In the first strategy only the weights of some newly added layers are optimized during training, while in the second strategy all the weights are optimized for the new task. Here, we consider fine-tuning as a more effective strategy that outperforms feature extraction and achieves better performance. As our pretrained networks explicitly require an RGB-input, we assigned identical values to the R, G, and B channels. Since the CT images in the two datasets have varying spatial sizes, the images needed to be scaled to match the target input size. One strategy to unify images with different aspect ratios involves stretching or excessive cropping. We opted for a different, less violating procedure and embed the image into a fixed-sized canvas. The aspect ratio of the original image was not altered and padding was applied to match the target shape.

## 5. Experiments and Results

This section presents our experimental setup and extensive experiments to show the efficacy of our fine-tuned networks. First, we describe the CT image datasets. Second, we state the experimental settings and performance evaluation metrics. Third, we discuss the results of different models on each dataset. Finally, we provide visual explanations to facilitate interpreting the decisions made by the models and to show their ability to localize the COVID-19-associated regions.

### 5.1. Datasets

**Figure 7:**
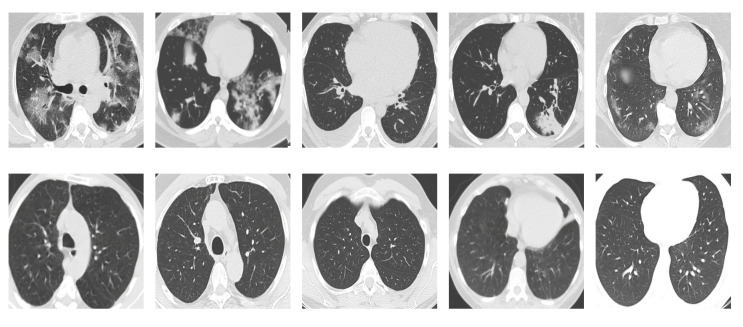
Examples of chest CT scans from the SARS-CoV-2 CT dataset. The first row represents CT scans diagnosed with COVID-19, whereas the second row represents non-COVID-19.

**SARS-CoV-2 CT Scan dataset** [53]: The dataset was collected from hospitals of Sao Paulo, Brazil, with a total of 2482 CT scans acquired from 120 patients of both genders. It is composed of 1252 scans for patients infected with SARS-CoV-2, and 1230 scans for patients infected with other lung diseases. The CT scans have varying spatial sizes between 104×119 and 416×512, and are available in PNG format. CT scans from this dataset are shown in Figure 7.

**Figure 8:**
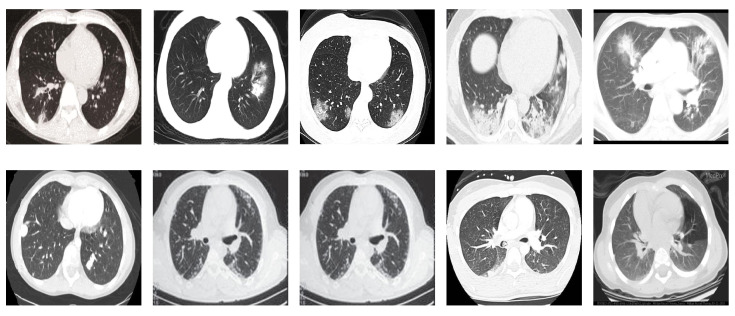
Examples of chest CT images from the COVID19-CT dataset. The first row represents CT images diagnosed with COVID-19, whereas the second row represents non-COVID-19 cases, but other lung diseases.

**COVID19-CT dataset** [54]: The dataset consists totally of 746 CT images. There are 349 CT images of patients with COVID-19 and 397 CT images showing non-COVID-19, but other pulmonary diseases. The positive CT images were collected from preprints about COVID-19 on medRxiv and bioRxiv, and they feature various manifestations of COVID-19. Since the CT images were taken from different sources, they have varying sizes between 124×153 and 1485×1853. Figure 8 shows example CT images from the COVID19-CT dataset.

### 5.2. Experimental Settings

To assess the performances of our models, we performed stratified K-fold cross-validation with K=5 to keep the distribution of the two classes consistent in each fold. The final performances of the models were computed by averaging the obtained values from the five networks on their test folds respectively.

Data augmentation methods were implemented to effectively increase the number of training samples for improved generalization. Affine transformations such as rotation and shearing turned out to have a worsening effect on performance, so we excluded this type of augmentation. More augmentation steps include cropping, adding blur with a probability of 25%, adding a random amount of Gaussian noise, changes in brightness and contrast, and random horizontal flipping. Finally, the images were normalized using the mean and standard deviation of the ImageNet dataset.

We followed a set of optimization configurations for all deep networks. The networks were optimized by applying the LAMB optimizer on a binary cross-entropy loss. The initial learning rate was set to 0.0003 and was scheduled to decrease according to the following steps: epoch 50: 0.0001, epoch 70: 0.00003, epoch 80: 0.00001, epoch 90: 0.000003. We used a batch size of 32 and we applied a high weight decay of 1 for regularization. The networks were implemented using the PyTorch framework and were trained for 100 epochs on a PC with Intel(R) Core(TM) i7-3770 CPU, 8 GB RAM, and Nvidia GTX 1080 GPU.

### 5.3. Evaluation Metrics

We consider different performance evaluation metrics for evaluating our models. For each model we count the number of predicted cases as true positives (TP), true negatives (TN), false positives (FP), and false negatives (FN). Then, we compute the following metrics.

(1) $$Accuracy=\frac{\mathrm{TP}+\mathrm{TN}}{\mathrm{TP}+\mathrm{TN}+\mathrm{FP}+\mathrm{FN}}$$

(2) $$Precision=\frac{\mathrm{TP}}{\mathrm{TP}+\mathrm{FP}}$$

(3) $$Sensitivity=\frac{\mathrm{TP}}{\mathrm{TP}+\mathrm{FN}}$$

(4) $$Specificity=\frac{\mathrm{TN}}{\mathrm{TN}+\mathrm{FP}}$$

(5) $$F1-score=\frac{2\times \mathrm{TP}}{2\times \mathrm{TP}+\mathrm{FP}+\mathrm{FN}}$$

### 5.4. Results and Discussion

Here, we present and discuss the results for detecting COVID-19 on the considered CT image datasets using our fine-tuned deep networks. We report the quantitative results along with the confusion matrices for every single architecture of the adopted networks.

Table 2 summarizes the average values of evaluation metrics achieved by our different networks on each CT image dataset. All values are given in percentages and the best results are written in bold. We also compare our results with the previously obtained results from the literature when applicable. Generally, we observed some performance differences between the results from the SARS-CoV-2 CT and the COVID19-CT datasets. We also observed the superiority of our obtained models compared with the similar models from the recently published work.

**Figure 9:**
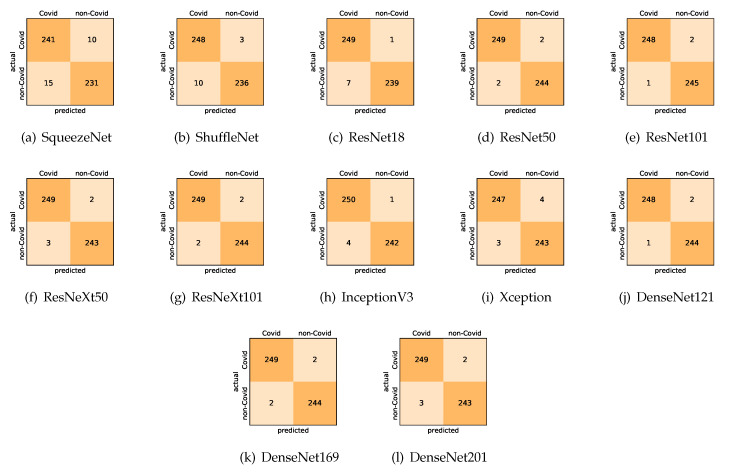
Confusion matrices for the different deep CNN models. These results are the average counts of the five models obtained by stratified 5-fold cross-validation on the SARS-CoV-2 CT dataset.

On the SARS-CoV-2 CT dataset, ResNet101 achieves the best overall performance with respect to almost all evaluation metrics, with an average accuracy and F1-score of 99.4%. The model also achieves an average sensitivity rate of 99.1% indicating that, on average, only two COVID-19 images are falsely predicted as negatives. It is also powerful enough to correctly identify all non-COVID-19 cases with only one false positive resulting a specificity rate of 99.6%. The highest sensitivity score of 99.8% is achieved by the InceptionV3 model, where only one COVID-19 image is falsely predicted as negative on average. The SqueezeNet model obtains the lowest performance with respect to all evaluation metrics with a fairly acceptable average accuracy and sensitivity scores of 95.1% and 96.2%, respectively. Compared with SqueezeNet, the ShuffleNet architecture obtains a satisfactory performance with approximately 2% improvement on average for all metrics. Although the results obtained by these models are inferior compared with the rest of models, but they are more computationally efficient, which matches their main objective designs of balancing the computational costs and their performance at visual recognition tasks. Comparing the various variants of ResNet and DenseNet, we observe that the deeper variants from each architecture yield a slightly better performance. Moreover, the deeper ResNet101 and ResNeXt101 show a marginal gain in performance compared with their shallower counterparts. The details about class-wise results for each model are summarized in the confusion matrices in Figure 9.

It is worth mentioning that on the SARS-CoV-2 CT dataset the inter-fold variations are minimal and usually below one percent, showing the robustness of our fine-tuning strategy. For some of the architectures like the DenseNet variants we observe a larger confidence interval than their actual differences in recognition performance. This means that the DenseNets and the deeper ResNet variants share a very similar performance and are almost indistinguishable from each other. Overall, the results obtained by our models are better than the recently published ones even when using the same network architectures. We attribute this to the better transferability of the learned features when applying our fine-tuning strategy. More specifically, the combination of the canvas approach to empirically choose the suitable input size for each architecture, and using larger input resolution than the standard CNN architectures. The chosen input size allows preserving the image ratios and tiling the network filters appropriately across the input volume, whereas the larger input size allows conveying more information.

On the COVID19-CT dataset, the overall performance with respect to all evaluation metrics is inferior to that on the SARS-CoV-2 dataset. This can be attributed to the cross-source heterogeneity of the CT images in the dataset. The non-COVID-19 CT images were taken from different sources and show diverse findings which pose difficulty to distinguish between COVID-19 and other findings associated with lung diseases due to the potential overlap of visual manifestations (see Figure 8). Another reason is that the CT images in the COVID19-CT dataset show strong variations in contrast, variable spatial resolution, and other visual characteristics which could affect the model’s ability to extract more discriminative and generalizable features.

**Figure 10:**
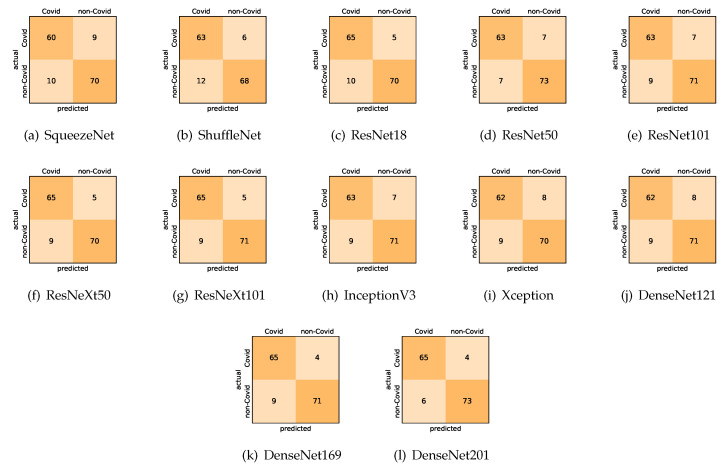
Confusion matrices for the different deep CNN models. These results are the average counts of the five models obtained by stratified 5-fold cross-validation on the COVID19-CT dataset.

Our models achieved fairly good performance compared with the recently published work using the exact network architectures. Here, we see that DenseNet201 outperformed all other architectures. The model achieved average accuracy and sensitivity scores of 92.9% and 93.7%, respectively. It also identified all COVID-19 cases, with only four images, on average, being falsely predicted as non-COVID-19. DenseNet169 achieved the second best average accuracy of 91.6% and a very high sensitivity identical to the best model. The DenseNet121 and Xception models achieved nearly identical results with respect to all evaluation metrics. We can observe that small networks such as ResNet18 achieved comparable results to those of other deeper models, whereas the SqueezeNet and ShuffleNet models performed at a similar level of accuracy. The variants of the ResNeXt models have comparable results and performed as well as the different ResNet variants. For detailed analysis and full understanding of the numbers of correct and misclassified cases for each individual model, see the confusion matrices presented in Figure 10.

It is also worth mentioning that for the COVID19-CT dataset the inter-fold variations grow substantially due to the small size of the dataset. During the stratified 5-fold cross-validation, the training set consists of about 600 images only, and the test fold has less than 200 images, which has to produce statistical fluctuations. Metrics considering the overall performance, such as the accuracy, have less inter-fold variation. However, we observed stronger variations in metrics that tested the bias towards one of the classes, such as the specificity. The standard deviation of the specificity indicates that the different folds tend to encourage the model to focus more on COVID-19 or more on non-COVID-19 cases. This phenomenon occurs even though for a stratified 5-fold cross-validation the distribution of classes in each fold represents the class distribution of the entire dataset, and it seems to originate from the small number of images only.

### 5.5. Visual Explanations

This subsection provides visual explanations to make our models more transparent. We start with a 2D projection of the learned features using t-SNE [14], and then present the localization maps for highlighting the COVID-19-associated regions using Grad-CAM [15].

#### 5.5.1. The t-SNE Visualization

**Figure 11:**
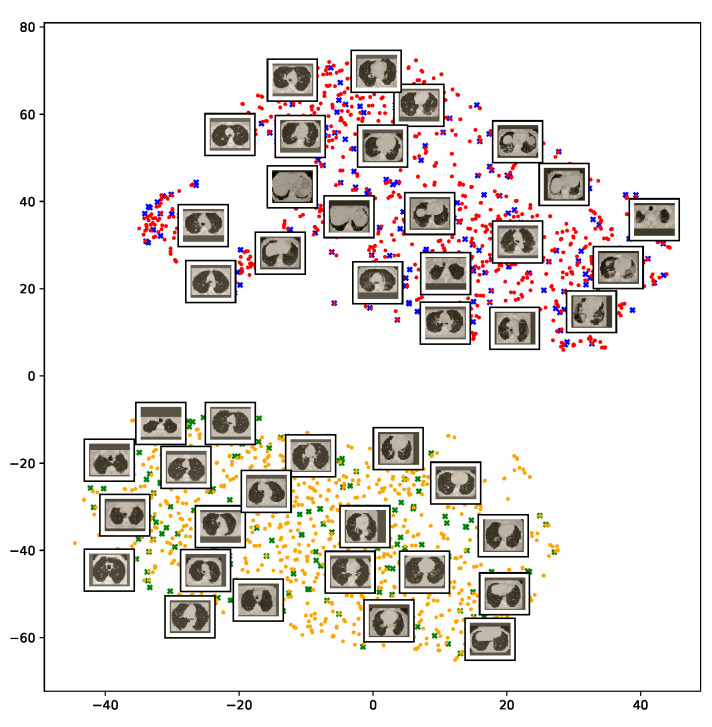
Visualization of the t-SNE embeddings for the entire SARS-CoV-2 CT dataset. We clearly see two different clusters representing COVID-19 (red for training and blue for test samples) and non-COVID-19 (yellow for train and green for test samples) classes.

To understand how the deep neural networks represent the CT images in the high-dimensional feature space, we applied the t-SNE algorithm to visualize these features. For each image in the SARS-CoV-2 dataset we first extracted the 2048-dimensional feature vector from the penultimate layer of the InceptionV3 model. Next, we applied t-SNE to map the features onto a 2D space and then visualize the embeddings of training and test representations. Figure 11 clearly shows two well-separated clusters of the CT images of COVID-19 and non-COVID-19 classes. The clear and wide margin between the two classes shows how nicely the CT images are separated in feature space. This indicates that the distributions of training and test features are quite similar to each other, indicating good generalization capabilities of our models.

**Figure 12:**
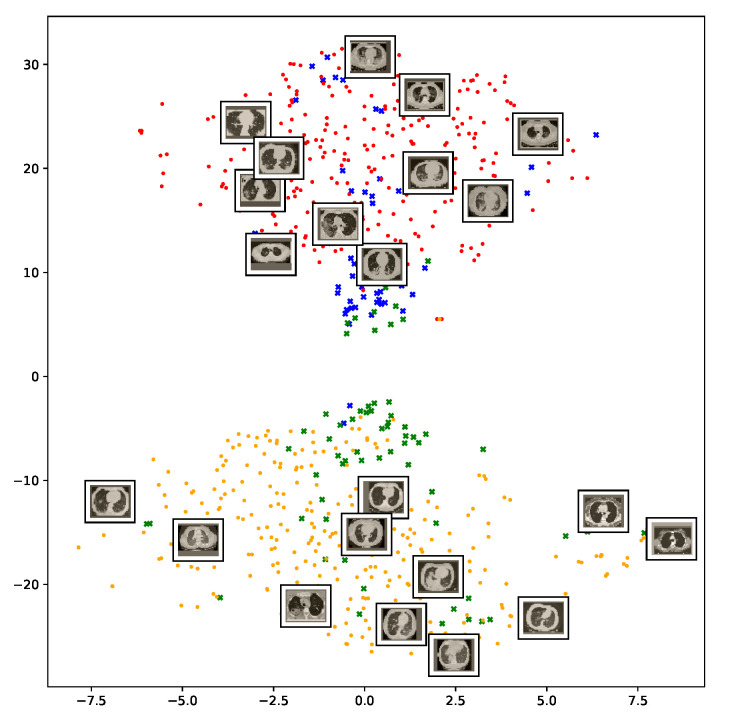
Visualization of the t-SNE embeddings for the entire COVID-19 CT dataset. As in Figure 11, we can see two different clusters representing COVID-19 and non-COVID-19 classes.

We also repeat the same procedure for the COVID19-CT dataset. The feature vectors are extracted from the penultimate layer of the DenseNet169 model. The length of the feature vectors is 1664 dimensions. We again apply t-SNE to map the features onto a 2D space to explore and visualize them. Figure 12 shows two clusters representing CT images for the COVID-19 and non-COVID-19 classes. Even though the classes are fairly distinguishable with a clear decision boundary, however, we can see that some CT images are misclassified, and more specifically the non-COVID-19 CT images from the test set.

#### 5.5.2. The Grad-CAM Visualization

**Figure 13:**
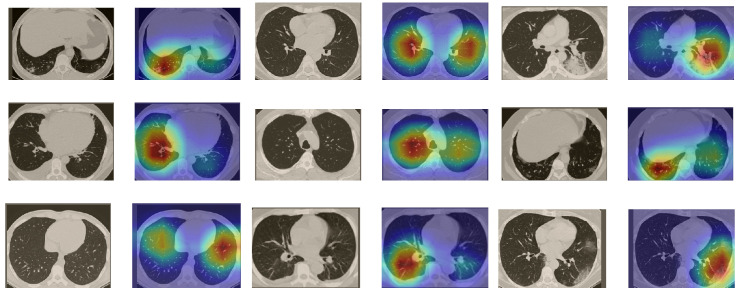
Grad-CAM visualizations for examples of CT images from the SARS-CoV-2 dataset. Our InceptionV3 model correctly classified them as COVID-19 and localized the most relevant regions used for its decision. The first, third, and fifth columns show CT images with COVID-19 findings, whereas the second, fourth, and sixth columns represent their corresponding localization maps generated by Grad-CAM.

In order to make our models more transparent and visually interpret the results, we provide the Grad-CAM localization maps generated by using different models. We utilized the CT images from COVID-19 class from the test set for each dataset, and highlight the important regions considered for the prediction. Figure 13 shows examples of CT images from the SARS-CoV-2 CT dataset and their localization maps. Interestingly, in most of the cases, our InceptionV3 model correctly classified them as COVID-19 cases and highlighted the regions of abnormalities in the CT scans, which are important for the model’s decision.

**Figure 14:**
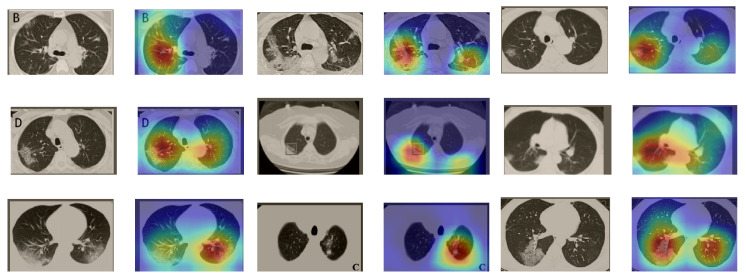
Grad-CAM visualizations for sample CT images from the COVID19-CT dataset. Our DenseNet169 model correctly classified them as COVID-19 cases and highlighted the most relevant regions, as shown in the corresponding localization maps.

In a similar way, we considered classifying the test CT scans from the COVID-19 dataset by the DenseNet169 model, and highlight the important regions considered for predictions. We present samples of CT images and their localization maps in Figure 14. We can also see that our model is capable of detecting the COVID-19-related regions as annotated (small square in some images) by expert radiologists.

**Figure 15:**
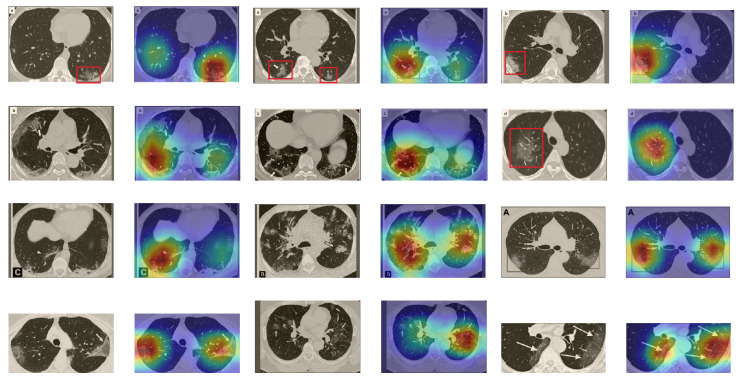
Examples of CT images taken from these two publications [61,62]. The CT images were correctly classified as COVID-19 cases, and the abnormal regions are accurately detected as in the localization maps.

A wide variety of typical and atypical chest CT abnormalities of COVID-19 patients have been reported in various studies [61,62]. In order to investigate the ability of our models to identify COVID-19 cases outside the considered datasets and localize their CT findings, we tested our models on external CT images extracted from these two publications, as they feature typical findings of COVID-19 pneumonia marked by specialists. To make sure that none of the extracted images were unintentionally included in our datasets, specifically the COVID19-CT dataset, we used the model trained on the SARS-CoV-2 dataset. First, the InceptionV3 model was used to classify the extracted CT images. The model was able to correctly classify the given CT images as COVID-19. Second, in order to interpret the model’s generalization capabilities, we applied the Grad-CAM technique to visualize the regions of abnormalities that are considered. By assessing the different CT images in Figure 15, we can see that the model accurately localizes the disease-related regions. Even more interesting is the fact that the model ignores any specific marks in the images such as the letters and only localizes the COVID-19-related regions. These further experiments prove the success of our models to learn distinguishable visual features related to COVID-19, and to correctly classify CT images outside the datasets on which they are trained and tested.

**Figure 16:**
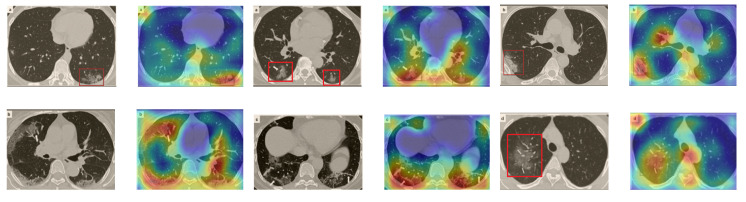
Grad-CAM visualizations for the same CT images in the first two rows of Figure 15. The CT images were correctly identified by SqueezeNet as COVID-19 cases with relevant localization of the disease-related regions.

In order to investigate the capacities of the different models to accurately localize identical or similar important regions, we employed the model with the lowest accuracy, i.e., SqueezeNet, which was fine-tuned on the SARS-COV-2 dataset to classify the unseen CT images taken from [61,62]. Moreover, we provide the Grad-CAM localization maps for a direct comparison. Figure 16 illustrates CT images, which represent the first two rows in Figure 15, and their corresponding localization maps. For most of the cases, both models looked exactly at similar regions representing the COVID-19 manifestations. However, for some images, SqueezeNet looked at irrelevant regions, such as the top left corner.

**Figure 17:**
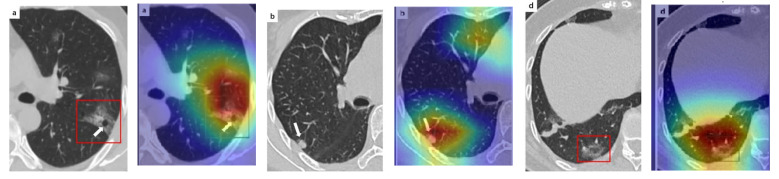
Example of annotated CT scans with different manifestations of COVID-19 taken from [61], and their corresponding localization maps. Our models were able to identify them as COVID-19 cases and accurately localize their COVID-19-associated regions.

Although we trained our models using CT images where both lungs are visible in the scans, we tested them on some external CT scans where only one lung is visible. The CT scans were extracted from the paper [61] and show different CT manifestations of COVID-19 marked by red squares or white arrows. Our models were able to classify them correctly as COVID-19 cases. Intriguingly, when applying Grad-CAM we can see from Figure 17 that all regions of abnormalities are accurately localized. This also proves the potential of our models to detect COVID-19 abnormalities in CT images outside the dataset used for training.

**Figure 18:**
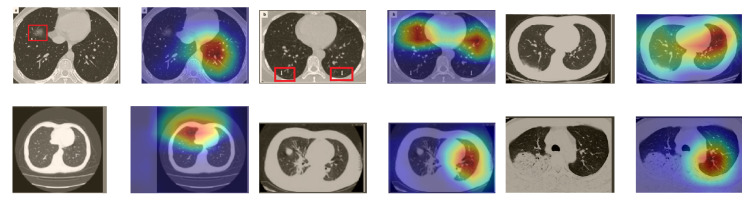
CT scans and their Grad-CAM localization maps showing cases in which the model failed to localize the most relevant COVID-19 regions.

For a comprehensive analysis of our visual explanations, we include some cases wherein the models failed to localize the exact COVID-19-associated regions. Figure 18 illustrates examples of CT scans that were correctly identified by our models as COVID-19 cases, but with which our models failed to localize the most relevant regions associated with COVID-19 as marked in some CT images. These failure behaviors can be attributed in some cases to focusing on similar findings or subtle density of manifestations found in the early days of infection. Nevertheless, in some cases the models only localized the findings in one lung and failed to highlight the disease-related regions in the other lung.

## 6. Conclusions

We proposed different deep learning based approaches for automated COVID-19 detection using chest CT images. The most advanced deep network architectures and their variants were considered, and extensive experiments were conducted on the two datasets with the largest numbers of CT images available so far. Moreover, we investigated different configurations and determined a custom-sized input for each network to achieve the best performance. The resulting networks showed significantly improved performance for detecting COVID-19. Our models achieved state-of-the-art performance with average accuracies of 99.4% and 92.9%, and sensitivity scores of 99.8% and 93.7% on the SARS-CoV-2 CT and COVID19-CT datasets, respectively. This indicates the effectiveness of our proposed approaches and the potential for using deep learning for fully automated and fast diagnosis of COVID-19. For better interpretability of the results, we provided visual explanations for decisions made by our models. We explored and visualized the learned features using the t-SNE algorithm, where the resulting visualizations showed well-separated clusters for COVID-19 and non-COVID-19 cases. We also assessed our models in localizing the abnormal regions in the CT images that are relevant to COVID-19. Moreover, we tested our models on external CT images from different publications, and our models were able to detect all COVID-19 cases and accurately localize the COVID-19-associated regions as marked by expert radiologists.

## Figures and Tables

**Table 1:** Characteristics of the deep CNN architectures considered for this work.

Model	Model Characteristics				
	Default Input Size	Custom Input Size	Layers	Parameters (M)	Model Size (MB)
SqueezeNet	227×227	335×255	18	0.73	3.0
ShuffleNet	224×224	321×225	51	0.34	1.5
ResNet18	224×224	349×253	18	11.17	44.8
ResNet50	224×224	349×253	50	23.51	94.3
ResNet101	224×224	349×253	101	42.50	170.6
ResNeXt50	224×224	349×253	50	22.98	92.3
ResNeXt101	224×224	349×253	101	86.74	347.9
InceptionV3	299×299	331×267	48	21.79	87.4
Xception	299×299	327×231	37	20.81	83.5
DenseNet121	224×224	349×253	121	6.95	28.3
DenseNet169	224×224	349×253	169	12.48	50.8
DenseNet201	224×224	349×253	201	18.09	73.6

**Table 2:** Performance comparison of different deep models for detecting COVID-19 using various evaluation metrics. The results are given in the form of mean and standard deviation scores. For a direct comparison, the results from the recently published work are included when applicable.

Dataset	Model	Evaluation Metrics				
		Accuracy	Precision	Sensitivity	Specificity	F1-Score
SARS-CoV-2 CT [53]	SqueezeNet	95.1±1.3	94.2±2.0	96.2±1.4	94.0±2.2	95.2±1.2
	ShuffleNet	97.5±0.8	96.1±1.4	99.0±0.2	95.9±1.5	97.5±0.8
	ResNet18	98.3±0.8	97.2±1.2	99.6±0.3	97.1±1.4	98.4±0.7
	ResNet50	99.2±0.3	99.1±0.5	99.4±0.5	99.1±0.5	99.2±0.3
	ResNet101	99.4±0.4	99.6±0.3	99.1±0.6	99.6±0.3	99.4±0.4
	ResNeXt50	99.1±0.5	99.0±0.5	99.3±0.5	98.9±0.6	99.1±0.5
	ResNeXt101	99.2±0.3	99.2±0.4	99.3±0.5	99.2±0.4	99.2±0.3
	InceptionV3	99.1±0.5	98.5±0.8	99.8±0.3	98.5±0.8	99.1±0.5
	Xception	98.8±0.6	99.0±1.0	98.6±1.1	98.9±1.1	98.8±0.6
	DenseNet121	99.3±0.3	99.4±0.2	99.2±0.5	99.4±0.2	99.3±0.3
	DenseNet169	99.3±0.5	99.4±0.6	99.3±0.5	99.3±0.7	99.3±0.4
	DenseNet201	99.2±0.2	99.0±0.4	99.4±0.2	98.9±0.4	99.2±0.2
	xDNN [53]	97.3	99.1	95.5	-	97.3
	DenseNet201 [38]	96.2	96.2	96.2	96.2	96.2
	Modified VGG19 [55]	95.0	95.3	94.0	94.7	94.3
	COVID CT-Net [56]	-	-	85.0±0.2	96.2±0.1	90.0±0.1
	Contrastive Learning [28]	90.8±0.9	95.7±0.4	85.8±1.1	-	90.8±1.3
COVID19-CT [54]	SqueezeNet	87.3±3.2	86.3±6.1	86.5±2.3	87.9±6.3	86.5±3.0
	ShuffleNet	87.9±2.6	84.5±2.5	90.8±3.9	85.4±2.7	87.6±2.8
	ResNet18	90.3±2.5	87.1±4.1	93.1±2.5	87.9±4.9	90.1±2.3
	ResNet50	90.8±1.9	90.2±5.0	90.0±3.6	91.4±5.0	90.1±1.9
	ResNet101	89.8±2.5	88.0±3.7	90.5±1.9	89.2±3.8	89.3±2.4
	ResNeXt50	90.6±2.2	87.4±3.6	93.4±3.4	88.2±4.4	90.3±2.2
	ResNeXt101	90.9±1.8	88.1±3.5	93.1±2.9	88.9±4.0	90.6±1.8
	InceptionV3	89.4±2.0	87.7±2.5	90.0±2.4	88.9±2.4	88.8±2.2
	Xception	88.5±2.6	87.3±2.7	88.3±4.7	88.7±2.9	87.7±2.9
	DenseNet121	88.9±1.2	87.6±2.6	88.8±1.4	88.9±2.9	88.2±1.0
	DenseNet169	91.2±1.4	88.1±2.5	93.7±1.2	88.9±2.7	90.8±1.4
	DenseNet201	92.9±2.2	91.3±2.2	93.7±3.4	92.2±2.2	92.5±2.4
	DenseNet169 [54]	83.0	-	-	-	81.0
	Decision function [57]	88.3	-	-	-	86.7
	ResNet101 [58]	80.3	78.2	85.7	-	81.8
	DenseNet121+SVM [59]	85.9±5.9	-	84.9±8.4	86.8±6.3	-
	DenseNet169 [60]	87.7±4.7	90.2±6.0	85.6±6.7	-	87.8±5.0
	Contrastive Learning [28]	78.6±1.5	78.0±1.3	79.7±1.4	-	78.8±1.4
